# The Short-Term Effects of Exercise on Intraocular Pressure, Choroidal Thickness and Axial Length

**DOI:** 10.1371/journal.pone.0104294

**Published:** 2014-08-29

**Authors:** Jie Hong, Hui Zhang, Debbie S. Kuo, Huaizhou Wang, Yanjiao Huo, Diya Yang, Ningli Wang

**Affiliations:** 1 Beijing Tongren Eye Center, Beijing Ophthalmology & Visual Sciences Key Laboratory, Beijing Tongren Hospital, Capital Medical University, Beijing, China; 2 Department of Ophthalmology, University of California San Francisco School of Medicine, San Francisco, California, United States of America; Wayne State University, United States of America

## Abstract

**Purpose:**

To explore ocular changes in healthy people after exercise.

**Methods:**

Twenty five volunteers underwent exercise for 15 minutes on a treadmill. Measurements of choroidal thickness, intraocular pressure (IOP), ocular biometry, and blood pressure were taken before and after exercise. Enhanced Depth Imaging optical coherence tomography (EDI-OCT) was used to measure choroidal thickness at the fovea. Intraocular pressure (IOP) was measured by Goldmann applanation tonometry. Ocular biometric measures were collected using A scan ultrasound. Blood pressure was measured concurrently with the acquisition of the scans.

**Results:**

Twenty five volunteers (25 eyes) with a mean age of 25.44±3.25 years were measured. There was a significant increase in systolic and diastolic pressure after exercise (P<0.05). The IOP showed a significant decrease after exercise (P<0.05). However there was no significant difference in the mean choroidal thickness, ocular axial length, anterior chamber depth, lens thickness, or vitreous length before and after exercise measurements (P>0.05).

**Conclusion:**

There was a significant decrease in IOP from exercise without a change in choroidal thickness and ocular biometric measures. IOP and choroidal thickness were not correlated, suggesting that the IOP decrease from exercise is not due to changes in choridal thickness.

## Introduction

Exercise has been demonstrated to lead to changes in a range of ocular parameters. Previous studies have shown a reduction in IOP following different types and intensities of exercise [1], [2], and it has been suggested that all forms of physical exercise such as bicycling, walking and jogging, decrease IOP [3], [4]. In normal subjects, the intraocular pressure decreases during exercise proportional to the work load [5]. However, the mechanism of IOP reduction has not been fully defined.

Physical activity has also been found to cause changes in some ocular parameters such as ocular blood flow [6], [7], tonic accommodation [8], pupil size [9], anterior chamber angle [10] and retinal activity [11]. Some investigators have theorized that increased choroidal volume may be enrolled in the mechanism of IOP elevation [12]. However, the ability to image the choroid successfully using ultrasonography and optical coherence tomography (OCT) has not been possible until recently. Since the development of OCT based enhanced depth imaging (EDI-OCT) by Spaide and colleagues [13], [14], successful examination and measurement of choroidal thickness in healthy and disease states has become a promising new method. Given that exercise is known to alter IOP and choroidal blood flow, we were interested in investigating the influence of exercise on IOP, choroidal thickness, ocular biometrics including axial length (AXL) to determine whether IOP reduction is correlated with changes in the choroid.

## Methods

### Subjects

Twenty five healthy individuals (15 men and 10 women) with a mean age of 25.44±3.25 years (ages 20 to 32 years) without history of ocular or systemic disease and not on any medications were included in the study. One eye from each subject was randomly selected for analysis of IOP, choroidal thickness and ocular biometric measurements. This study was approved by the Ethics Committee of Beijing Tongren Hospital, Capital Medical University and all volunteers signed an informed consent before the start of the trial after explanation of the nature and possible consequences of the study. The research followed the tenets of the Declaration of Helskinki. As previous studies have indicated that choroidal blood flow regulation may be altered in chronic smokers, no regular cigarette smokers were included in the study [15]. Each subject also underwent a preliminary ophthalmologic examination before participating in the study to exclude any patients with pre-existing, undiagnosed ocular disease. Before the study, all volunteers had their choroids imaged. We selected subjects with the most clearly visualized choroid/scleral border for the study in order to have the most accurate measurements. Subjects were instructed to refrain from ingesting food or liquid in the 30 minutes prior to the experiment, as water loading has previously been found to influence AXL and IOP [16].

### Blood Pressure Measurements and Exercise Intensity Control

Blood pressure (BP) measurements were performed using an automated BP cuff. Resting BP was taken before exercise and post-exercise BP was taken immediately after exercise. All individuals exercised for 15 minutes on an electric treadmill. To standardize the intensity of the exercise between subjects, heart rate was monitored throughout the exercise task and subjects ran at an intensity that maintained their heart rate above 70% of their maximum heart rate, which was defined as 208 - (0.7*age). The speed of electric treadmill ranged from7–8 km/h for men and 6.5–7.5 km/h for women.

### IOP measurements

The IOP of both eyes were measured before exercise and again at 0, 10, and 30 minutes after exercise by Goldmann applanation tonometry. Each IOP measurement was performed at least twice and in cases where more than a 2 mmHg difference existed between the first two measurements, a third measurement was performed the mean of the two higher values was used in subsequent analysis.

### Choroidal thickness measurements

The method of obtaining EDI-OCT images has been reported previously [17]. Subfoveal choroidal thickness (SFCT) was measured using a spectral-domain OCT device (Spectralis: wavelength, 870 nm; Heidelberg Engineering Co., Heidelberg, Germany) with an enhanced depth imaging mode. Seven sections, each comprising 100 averaged scans, were obtained at an angle of 5°×20° in a rectangle centered on the fovea. The horizontal section running through the center of the fovea was selected for further analysis. Subfoveal choroidal thickness was defined as the vertical distance from the hyperreflective line of the Bruch's membrane to the hyperreflective line of the inner surface of the sclera. The measurements were performed using the Heidelberg Eye Explorer software (version 5.3.3.0; Heidelberg Engineering Co.).

In a previous study, subfoveal choroidal thickness measurements by EDI-OCT showed a high intra-observer reproducibility and interobserver reproducibility [17].

### Ocular biometric measurements

Ocular biometric measurements were collected using contact A scan ultrasound (Suoer, SW-2100, China). The anterior chamber depth (ACD), lens thickness (LT), vitreous length (VL) and axial length (AXL) of both eyes were measured. Measurements were collected according to manufacturer specifications with five repeated measurements collected from each subject at each measurement session.

### Statistics

The data were analyzed by a commercial analytical software program (SPSS 16.0; SPSS). A repeated measures ANOVA analysis was used to compare choroidal thickness at fovea, IOP and ocular biometrics. Correlation analysis was used to determine whether there was correlation between IOP and choroidal thickness. P<0.05 was considered to be statistically significant.

## Results

### IOP

**Figure 1:**
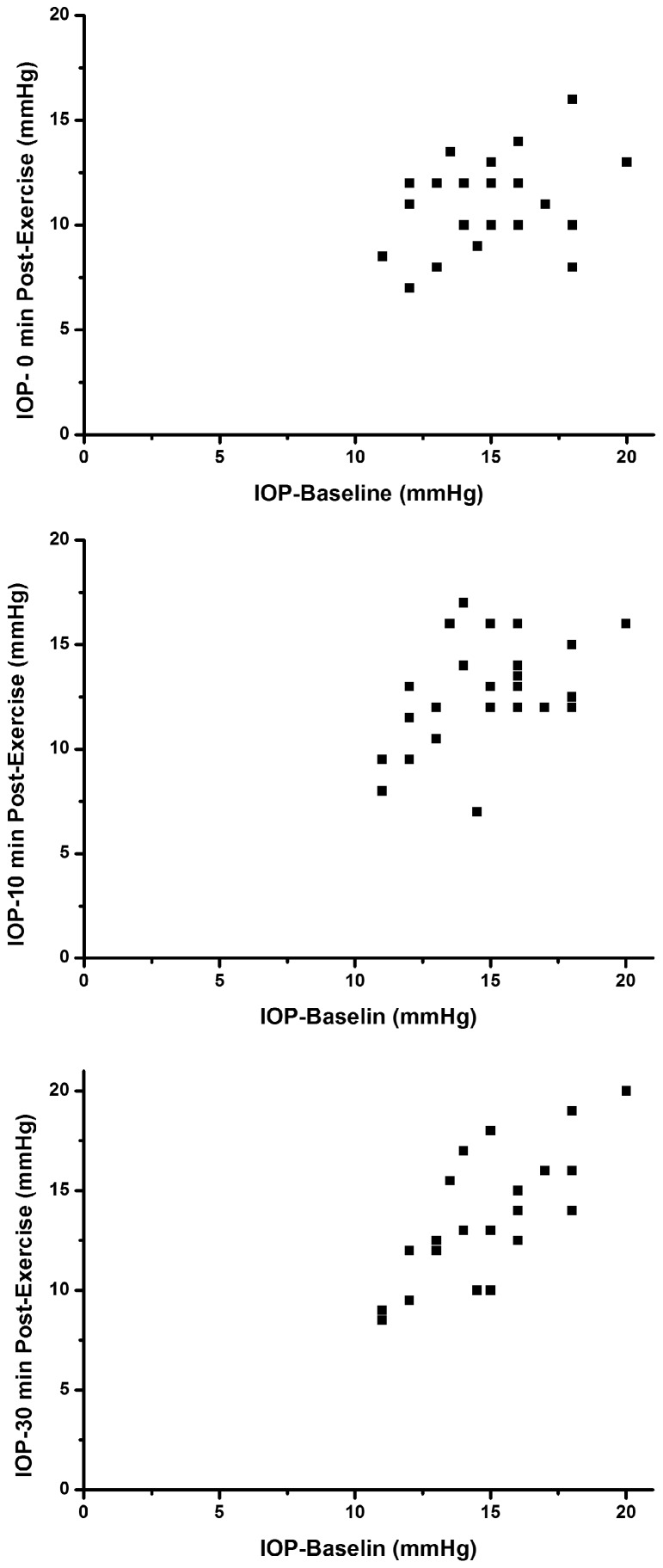
IOP scatterplot before and after exercise.

**Figure 2:**
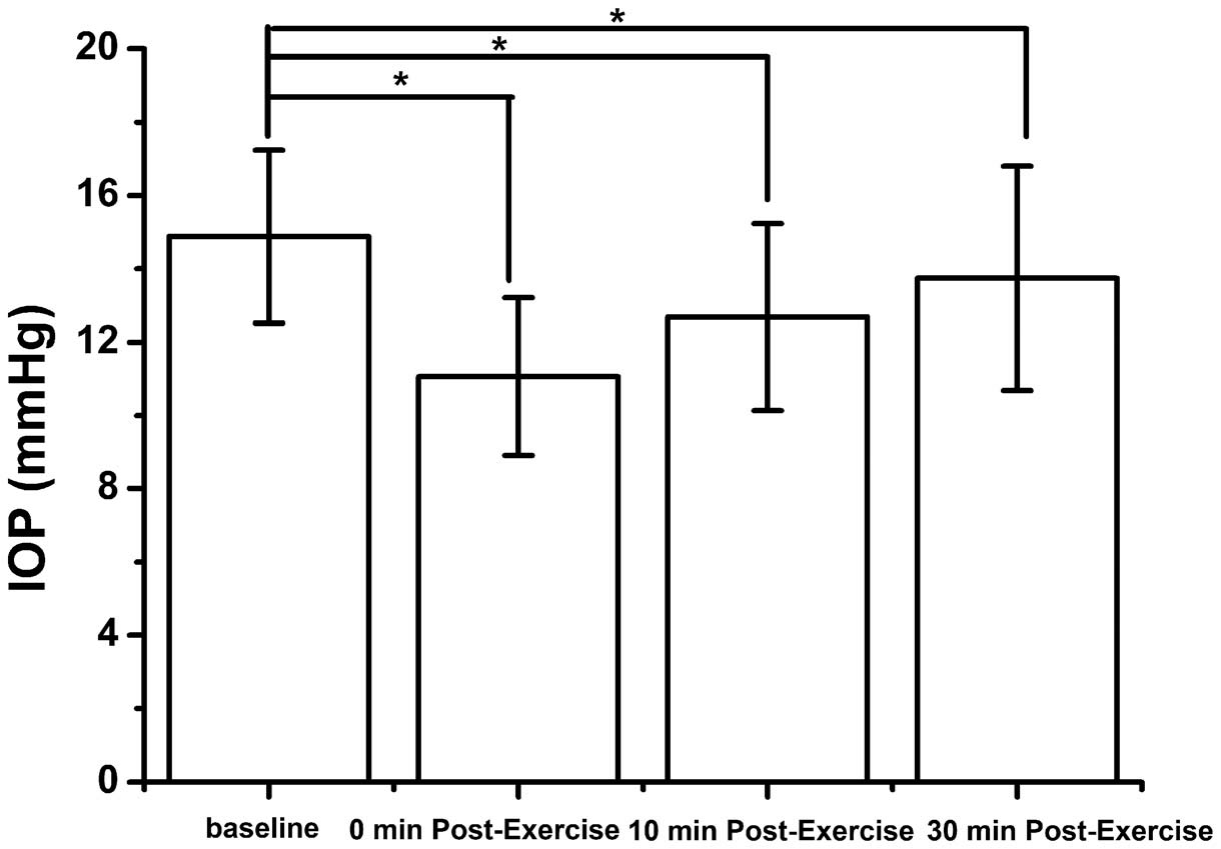
IOP before and after exercise. A significant decrease was observed before and after exercise (p<0.001).

Twenty five healthy volunteers (25 eyes) with a mean age of 25.44±3.254 years were studied. In this population of young adult subjects, 15 minutes of dynamic exercise was found to lead to significant changes in IOP, consistent with prior reports showing that exercise leads to IOP reduction (Table 1 and Figure 1, 2). The measurements of IOP were normally distributed. The mean IOP at baseline was 14.88±2.36 mmHg, which reduced on average to 11.06±2.15 mmHg immediately after exercise, 12.68±2.55 mmHg at 10 min and 13.74±3.06 mmHg at 30 min after exercise. Repeated measures ANOVA analysis revealed significant reductions in IOP following exercise (P<0.01).

**Table 1:** The Effects of Exercise on Intraocular Pressure and Choroidal Thickness.

(n = 25)	mean±SD at baseline	Mean±SD after exercise			P value
		0 min	10 min	30 min	
IOP(mmHg)	14.88±2.36	11.06±2.15	12.68±2.55	13.74±3.06	<0.001
CT(um)	346.82±58.74	341.63±55.71	335.54±51.14	342.61±55.22	0.101

IOP: intraocular pressure, CT: choroidal thickness.

### Choroidal thickness

**Figure 3:**
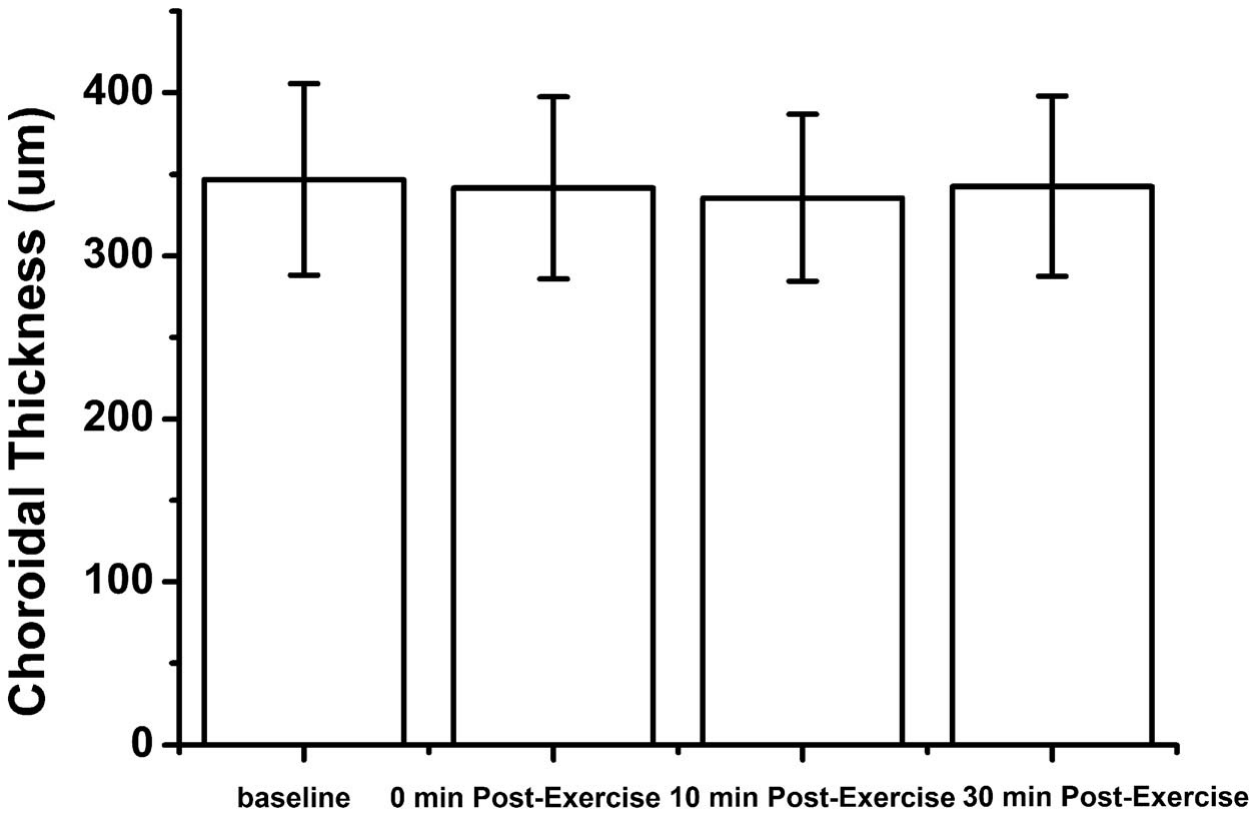
Subfoveal choroidal thickness before and after exercise. No significant change was observed before and after exercise.

Choroidal thickness measurements before and after exercise are shown in Table 1. The measurements of choroidal thickness were normally distributed. There was no significant change in choroidal thickness after exercise (P>0.05) (Figure 3). The average choroidal thickness before exercise was 346.82±58.71 um, which decreased to an average of 341.63±55.71 um immediately after exercise, 335.54±51.14 um at 10 minutes and 342.61±55.22 um at 30 minutes after exercise.

There was no significant correlation between between IOP and choroidal thickness (P = 0.253).

### Ocular biometric measures

**Figure 4:**
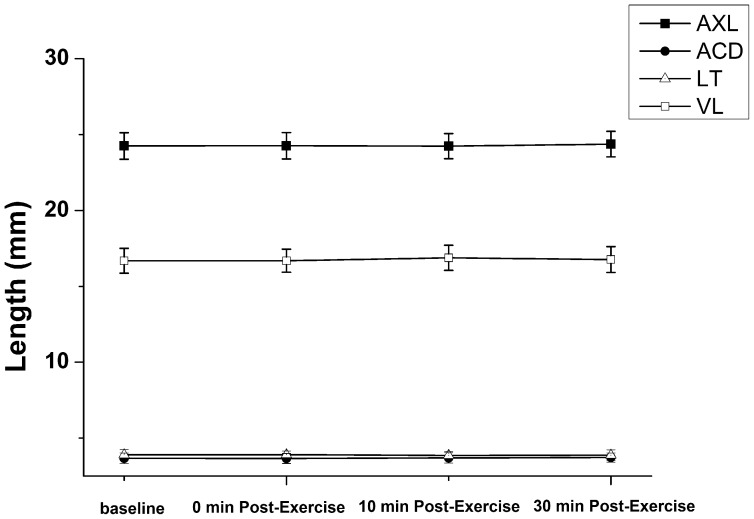
Ocular biometry before and after exercise. No significant differences were observed before and after exercise.

Prior studies have demonstrated changes in IOP and AXL following exercise [31]. To explore this relationship, we measured AXL, along with ACD, LT, VL, before and after exercise. The mean ocular biometric measures and their changes following exercise are displayed in Table 2. There were no significant differences in AXL, ACD, LT and VL after exercise (Figure 4).

**Table 2:** The Effects of Exercise on Ocular Biometry.

(n = 25)	mean±SD at baseline	Mean±SD after exercise			P value
		0 min	10 min	30 min	
AXL (mm)	24.25±0.87	24.26±0.87	24.24±0.83	24.37±0.84	0.95
ACD (mm)	3.67±0.35	3.66±0.33	3.69±0.34	3.72±0.30	0.40
LT (mm)	3.90±0.34	3.91±0.24	3.86±0.27	3.88±0.35	0.81
VL (mm)	16.70±0.82	16.69±0.76	16.68±0.83	16.77±0.85	0.56

AXL: axial length, ACD: anterior chamber depth, LT: lens thickness, VL: vitreous length.

### Blood pressure

Analysis of systemic hemodynamic parameters was shown in Table 3. The data revealed a significant increases in systolic, diastolic pressure and mean arterial pressure (MAP) following exercise (P<0.001). The changes in BP coupled with the reduction in IOP lead to significant increases in ocular perfusion pressure (OPP) following exercise (P<0.001). A mean increase in OPP of 10.47 mmHg was found immediately after exercise. The correlation between choroidal thickness and OPP (R = 0.096, P = 0.509), MAP (R = 0.134, P = 0.354) were statistically insignificant. The change in choroidal thickness also showed no significant correlation with the change of OPP (R = −0.106, P = 0.615) and MAP (R = −0.191, P = 0.360) after exercise. IOP was significantly correlated with OPP (R = −0.496, P<0.001), however the amount of decrease of IOP was not significantly correlated to amount of increase in OPP after exercise (R = 0.272, P = 0.188).

**Table 3:** The Effects of Exercise on Blood Pressure.

	mean±SD before	mean±SD after exercise (0 minute)	P value
SBP	113.80±12.01	129.00±18.82	<0.001
DBP	75.80±9.09	83.16±11.35	<0.001
MAP	88.47±9.40	98.44±12.73	<0.001
OPP	44.10±6.51	54.57±8.47	<0.001

SBP: systolic blood pressure, DBP: diastolic blood pressure, MAP: mean arterial pressure, OPP: ocular perfusion pressure.

## Discussion

Isometric exercise and dynamic exercise can both result in acute decreases in IOP [3], [18], [19]. Our data confirm the IOP lowering effect of dynamic exercise in a population of healthy young adults, consistent with previous studies. The underlying mechanism of IOP reduction during dynamic exercise has not been fully defined and the associated physical changes in the eye are just starting to be explored. In this study, we found no significant change in sub-foveal choroidal thickness and other ocular biometric measures with dynamic exercise. Furthermore, there was no correlation between IOP and choroidal thickness, which implies that the choroid is not etiology for IOP reduction after exercise.

In our population of young healthy volunteers, choroidal thickness was not significantly changed by exercise. This data is in agreement with that from Alwassia et al, [20] in spite of the different subjects and exercise intensity. Alwassia et al. studied patients with a mean age of 60.6±10.4 years who underwent cardiac exercise stress testing in the cardiovascular stress testing unit. Exercise was stopped as soon as patients reached a given heart rate. In our more strenuous exercise regimen, healthy individuals with a mean age of 25.44±3.25 years ran for 15 minutes on treadmill at 6.5–8 km/h for a total of 1.625 to 2 km, and continued running even after reaching 70% of their maximal heart rate. Previous publications have reported that the decrease in IOP was directly related to exercise intensity [3], [21], [22], [23], which raises the question whether other ocular changes such as effects on the choroid might also be dependent on exercise intensity and potentially missed without an adequate level of exercise. However our results and those of Alwassia et al. demonstrated exercise does not induce acute significant change in choroidal thickness, even at different exercise intensity levels and in different aged populations though this has not been formally studied.

Our investigation demonstrated no significant change in the thickness of the choroid after exhaustive exercise, despite significant change in BP, indicating an adaptation of vascular resistance due to vasoconstriction. The mechanism of this regulation is probably mediated by autoregulation or by the intricate neural innervation of the choroidal vessels [24]. The myogenic theory assumes that changes in transmural pressure are responsible for smooth muscle constriction in response to an OPP increase, in an effort to keep vessel wall tension constant [6]. Both sympathetic and parasympathetic nerves have been identified in the choroid [25], [26], [27] and could contribute to neural regulatory mechanisms in the choroid.

Our study demonstrates that exercise leads to a significant decrease in IOP but not in AXL, which is not consistent with the results of Read and Collins [28]. Read and Collins reported a decrease in AXL immediately after (mean change −17±12 um) and 5 min after exercise (mean change −7±8 um) that were significantly different to baseline (P<0.01), and a return to baseline AXL 10 min after exercise (mean change −3±9 um) [31]. However, we measured AXL before exercise, immediately after, 10 min and 30 min after exercise, and did not detect a difference. One possible explanation for this finding is that there was a change below the threshold of what we were able to detect with A-scan ultrasound. The maximal change in AXL following exercise detected by Read and Collins was only 0.07% of AXL (mean change −17±12 um) using the Lenstar 900 [31]. The resolution and precision of partial coherence interferometry is reported to be better than that of ultrasound-based methods [29], [30] and could account for the differences between our results. Interestingly, Read and Collins observed a trend for a small increase in CT (mean change of ∼3%) following exercise, but the changes in CT did not reach statistical significance. They presumed expansion of the choroid lead to an accompanying reduction in AXL. However, our data and that of Alwassia et al. do not support this theory [19].

Several studies have shown a decrease of AXL with lowered IOP, such as in the setting of trabeculectomy [31], [32] or after pharmacological treatment [33]. Although previous studies found a significant association between the changes in AXL and IOP, other factors that might affect AXL cannot be ruled out. Francis et al. [34] evaluated axial eye length shortening after glaucoma surgery, but the structural integrity of eye might be considerably altered by filtering surgery. Leydolt et al. [35] used a suction cup causing mechanical stress and local deformation to the sclera to induce IOP changes, however, the use of the suction cup could cause deformation of the entire globe, resulting in axial eye length alterations independent of pressure change. The relationship between AXL and IOP remains to be better defined and further explored, but in our study we found that there was no relationship between the two variables in the setting of exercise.

Our results showed that there was no correlation between IOP and choroidal thickness, which implies that the choroid is not responsible for IOP reduction after exercise. Our findings are important because it may change our view regarding the relationship of choroidal thickness and IOP as previous studies have suggested a link between these two variables.
